# 1710. *Clostridioides difficile* Bundle Implementation Reduces Unnecessary Testing in Pediatric Patients

**DOI:** 10.1093/ofid/ofad500.1543

**Published:** 2023-11-27

**Authors:** Lydia Sietsema, Holly Maples, Michele Honeycutt, Bobby Boyanton, John Forbus, Jessica Snowden

**Affiliations:** Arkansas Children's Northwest, Springdale, Arkansas; University of Arkansas for Medical Sciences, College of Pharmacy, Little Rock, Arkansas; Arkansas Children's Hospital, Little Rock, Arkansas; Arkansas Children's Hospital, Little Rock, Arkansas; Arkansas Children's Hospital, Little Rock, Arkansas; University of Arkansas for Medical Sciences, Little Rock, Arkansas

## Abstract

**Background:**

Failure to follow proper guidelines can lead to inappropriate *Clostridioides difficile* infection (CDI) testing in pediatric patients, resulting in incorrect diagnoses and antibiotic overuse. The Infectious Diseases Society of America and Society for Healthcare Epidemiology of America recommend a 2-step testing algorithm incorporating restrictions based on age, exposure, and underlying conditions. With these recommendations, a CDI bundle was implemented to reduce unnecessary testing in pediatric patients. Outcomes were measured via National Healthcare Safety Network (NHSN) LabID reporting.

**Methods:**

An interdisciplinary team reviewed the CDI ordering process in a 24-bed community pediatric hospital with an Emergency Department (ED). Previous testing only used toxigenic *C. difficile* PCR without order restrictions. In September 2021, a CDI bundle was implemented, including a 2-step algorithm (toxigenic *C. difficile* PCR, toxin A/B immunoassay), a physician guidance pathway, and Epic order restrictions (Table 1).

Outcomes were measured by examining NHSN LabID data before and after bundle implementation. Inpatient and ED data were reported under the appropriate NHSN patient safety module. CDI rates were calculated by the number of infections/1000 inpatient days and number of infections/1000 ED encounters. There was a 12-month pre-implementation period (August 2020-July 2021), a 3-month implementation period (August 2021-October 2021), and a 12-month post-implementation period (November 2021-October 2022).

**Figure:**
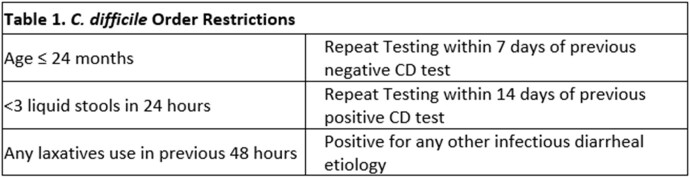

**Results:**

A statistically significant decrease in the CDI incidence was seen after CDI bundle implementation (P< 0.05 for both inpatient and ED using test of proportions). The average CDI incidence decreased from 2.24 to zero infections/1000 inpatient days (Figure 1) and from 0.58 to 0.02 infections/1000 ED encounters (Figure 2).

**Figure:**
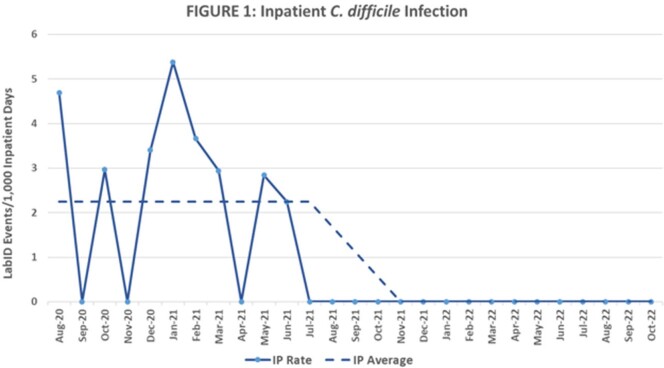

**Figure:**
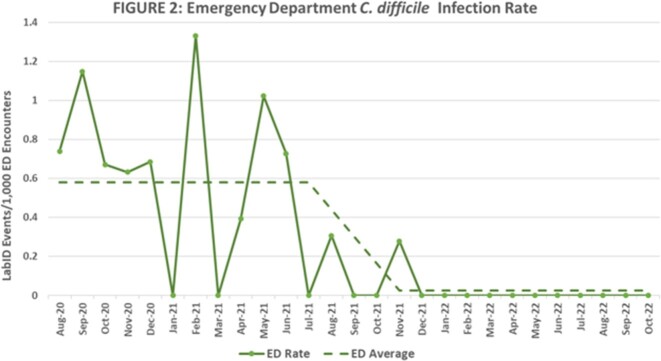

**Conclusion:**

CDI diagnostic bundle implementation effectively decreased the number of abnormal CDI test results, and positively impacted data reported to NHSN. Further analysis is warranted to delineate the impact of CDI bundle implementation on patient treatment, antimicrobial stewardship, and healthcare costs.

**Disclosures:**

**Jessica Snowden, MD, MHPTT**, Pfizer: Advisor/Consultant

